# Spin Dynamics of (Sc$_{1-x}$Lu$_{x}$)$_{3.1}$In Studied by Electron Spin Resonance

**DOI:** 10.1007/s00723-018-0987-0

**Published:** 2018-02-13

**Authors:** Archie Cable, Eteri Svanidze, Jessica Santiago, Emilia Morosan, Jörg Sichelschmidt

**Affiliations:** 1 Max Planck Institute for Chemical Physics of Solids, Dresden, Germany; 2 Department of Physics and Astronomy, Rice University, Houston, TX 77005 USA

## Abstract

The electron spin resonance (ESR) of conduction electrons is reported for the weak itinerant ferromagnet Sc$_{3.1}$In which, upon chemical substitution with Lu, shows a suppression of ferromagnetic correlations. A well-defined ESR lineshape of Dysonian type characterizes the spectra. The ESR linewidth, determined by the spin dynamics, displays a broad minimum only for the Sc$_{3.1}$In compound. We discuss the results using the mechanism of exchange enhancement of spin-lifetimes.

## Introduction

The weak itinerant ferromagnet Sc$_{3.1}$In recently came into focus, because a quantum critical point (QCP), accompanied by non-Fermi liquid behavior, was reported to occur upon chemical substitution of the Sc site by Lu [1]. The QCP was found to occur at a critical composition $x_{\mathrm{c}}=0.035\pm 0.005$ as evidenced by an analysis of the magnetization data as well as muon-spin relaxation measurements. This was a remarkable observation as Sc$_{3.1}$In is a rare example of an itinerant ferromagnet composed of non-magnetic elements for which quantum critical behavior has been investigated. Close to $x_{\mathrm{c}}$, a logarithmic divergence of the specific heat and a linear temperature dependence of the resistivity indicate non-Fermi liquid behavior. The reduced crystallographic dimensionality (associated with quasi-1D Sc-In chains) favours strong spin fluctuations, which give rise to the magnetism, which is well described by a modified Curie–Weiss-*like* law for weak itinerant ferromagnets [2].

For investigating the spin dynamics of itinerant magnets, the technique of electron spin resonance (ESR) has proven to be a powerful tool [3–5], since the exchange enhancement of spin-lifetimes enables strong and narrow lines [6, 7]. Early ESR results on Sc$_{3.12}$In reveal a spin resonance with a lineshape typical for itinerant resonant spins [8]. An investigation of the ESR in (Sc$_{1-x}$Lu$_{x}$)$_{3.1}$In should, in principle, enable us to observe how changing the ferromagnetic correlations with Lu substitution influences the linewidth.

## Experimental

We used polycrystalline samples of (Sc$_{1-x}$Lu$_{x}$)$_{3.1}$In with $x=0$, 0.02, 0.025, 0.03, and 0.04. Their preparation along with their magnetic, transport, and thermodynamic properties is reported in Ref. [1]. The composition Sc$_{3.1}$In yields the highest paramagnetic moment $\mu _{\mathrm{PM}}=1.3\mu _{\mathrm{B}}$/F.U. and a Curie temperature $T_{\mathrm{C}}=4.5$ K. It was shown [1] that a non-mean field description was most accurate for describing the magnetism in this compound, which also resulted in the highest $T_{\mathrm{C}}$ at the Sc:In = 3.1:1 composition.

The ESR measurements were performed at X-band ($\nu =9.4$ GHz) and Q-band frequencies ($\nu =34.1$ GHz) using a commercial spectrometer together with a He-flow cryostat allowing for temperatures between 1.6 and 300 K to be considered. ESR probes the absorbed power *P* of a transverse magnetic microwave field as a function of a static and external magnetic field *B*. To improve the signal-to-noise ratio, we used a lock-in technique by modulating the static field, which yields the derivative of the resonance signal d*P*/d*B*.

To obtain the linewidth $\Delta B$ and the resonance field $B_{\mathrm{res}}$ of the measured ESR spectra, we utilized a metallic Lorentzian shape [5, 9]. This shape contains a dispersion-to-absorption ratio $\alpha$ that describes microwave dispersion from the skin effect but also mimics the more general shape for itinerant resonant spins (conduction electrons), the so-called Dysonian lineshape [10, 11]. This ”Dysonian” is crucially determined by the diffusion of the resonating spins through the microwave penetration depth $\delta$ of the conductive environment. The ratio between the average time of diffusion within $\delta$ and the time of electron spin relaxation, $T_{\mathrm{D}}/T_{2}$, is related to the asymmetry parameter *A* / *B* which is the ratio between the maximum and minimum of the d*P*/d*B* lineshape. $A/B=2.7$ refers to the local, stationary case with $T_{\mathrm{D}}/T_{2}\rightarrow \infty$ for which a dispersive Lorentzian shape is applicable.

For our samples and $\nu \le 34.1$ GHz, the smallest $\delta$ was about 1 μm at the lowest temperatures where $\rho \approx 20 \, \upmu \Omega {\mathrm cm}$ [1]. Thus, with a sample thickness of about 0.5 mm, the case of a ’thick metal plate’ in Dyson’s theory applies.

## Results and Discussion

**Fig. 1:**
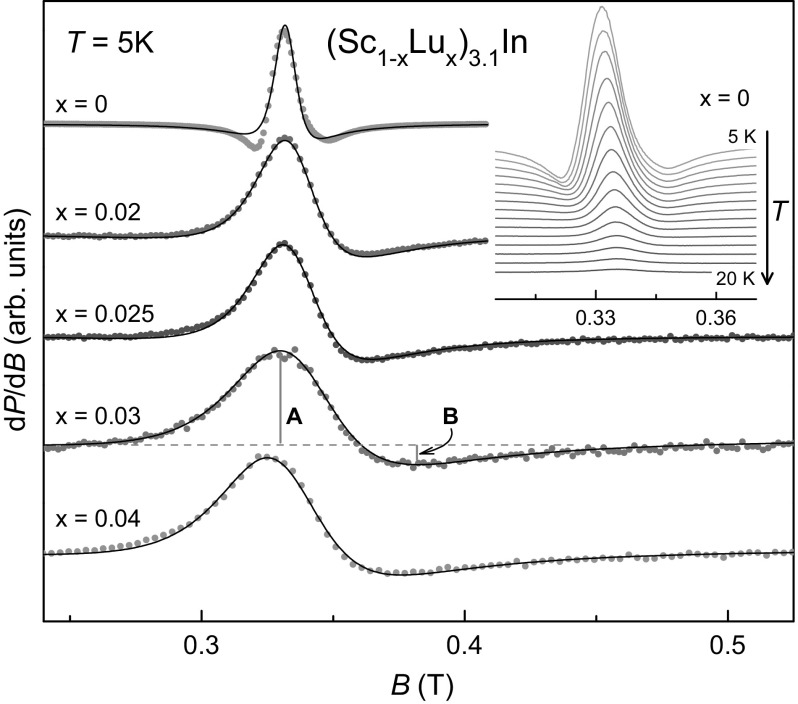
ESR spectra of (Sc$_{1-x}$Lu$_{x}$)$_{3.1}$In. The solid lines denote a fit by a metallic Lorentzian function, which yields the linewidth and resonance field. The asymmetry parameter $A/B=5\pm 0.7$ for all *x*. Inset displays the temperature evolution of the $x=0$ spectra

The previously reported ESR results obtained on polycrystalline plates of Sc$_{3.12}$In [8] could be nicely reproduced with our samples of (Sc$_{1-x}$Lu$_{x}$)$_{3.1}$In for $x=0$. Figure 1 shows the evolution of the ESR lines with Lu for $T=5$ K. In terms of a Dyson lineshape analysis, the line asymmetry *A* / *B* shows only small changes: $A/B=5\pm 0.7$ for all investigated Lu concentrations. Assuming the ’thick metal plate’ case in Dyson’s theory, these *A* / *B* values mean that $0.24<T_{\mathrm{D}}/T_{2}<0.55$, values typical for itinerant resonant spins.

The pure sample ($x=0$) shows an additional negative lobe which develops with decreasing temperature, as shown in the inset of Fig. 1. This spectral feature was also observed previously [8] and cannot be described well by a metallic Lorentzian (solid line in Fig. 1, $\alpha =9$, i.e., a shape with a huge dispersion part, see discussion below). It shows a weak dependence on the polycrystal orientation in the magnetic field and, hence, might originate from demagnetisation effects in the proximity of magnetic ordering. It is worth noting that a very similar lineshape was recently observed in the ESR investigation of localized Nd$^{3+}$ ions in the semimetal YBiPt, where highly mobile conduction electrons in the skin depth lead to the metallic and diffusive Dysonian lineshape [12].

The investigated samples with different Lu concentration have comparable penetration depths (because of their similar resistivity [1]). Hence, with Lu substitution, the increasing linewidth should result in more symmetric lines, i.e., *A* / *B* is expected to decrease. This is because a line broadening with Lu substitution means a decrease of $T_{2}$ and an increase of $T_{\mathrm{D}}$ (assuming that a line broadening is related to the transport scattering process *via* spin–orbit coupling) and, hence, an increase of $T_{\mathrm{D}}/T_{2}$ (decrease of *A* / *B*) should be observed [10]. Unfortunately, within the possible accuracy in determining *A* / *B*, we could not resolve clear changes in $T_{\mathrm{D}}/T_{2}$ of the itinerant resonant spins.

**Fig. 2:**
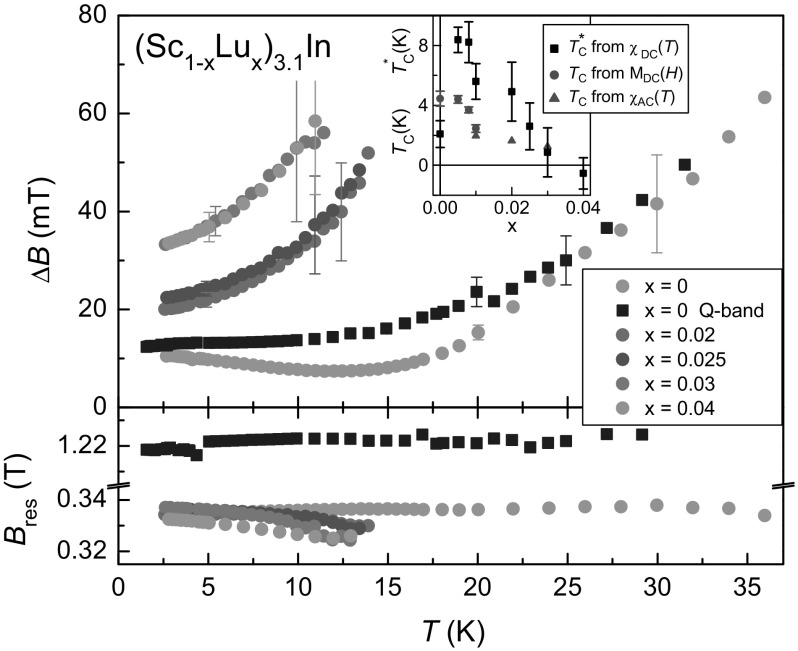
Temperature dependences of ESR linewidth $\Delta B$ and resonance field $B_{\mathrm{res}}$ of (Sc$_{1-x}$Lu$_{x}$)$_{3.1}$In. X- and Q-band data are denoted by filled circles and squares, respectively. Inset: phase diagram, showing the ferromagnetic ordering temperature $T_{\mathrm{C}}$ and the Curie–Weiss-*like* temperature $T^{*}_{\mathrm{C}}$, obtained by the indicated methods (adapted from [1])

We used a metallic Lorentzian line fitting (solid lines in Fig. 1) to extract $\Delta B$ and $B_{\mathrm{res}}$. To optimise the Lorentzian fit to the Dysonian lineshape, we used the parameter $\alpha$ (which in the case of local spins would be a measure of the microwave dispersion). It varies for different *x* values ($\alpha =9, 2, 1.8, 1.8, 1.8$ for $x=0$, 0.02, 0.025, 0.03, and 0.04, respectively), but we kept it constant to describe the temperature dependences of $\Delta B$ and $B_{\mathrm{res}}$, which are shown in Fig. 2. The large $\alpha$ value for $x=0$ provides the best fit of the pronounced negative lobe in the line shape.

For $x=0$, the published linewidth data, taken at $\nu =12$ GHz with $B_{\mathrm{res}}\approx 0.42$ T [8], are similar to our data taken at $\nu =9.4$ GHz with $B_{\mathrm{res}}\approx 0.33$ T but with a notable shift of the shallow minimum towards lower temperatures. Our $\nu =34$ GHz Q-band data ($B_{\mathrm{res}}\approx 1.22$ T) do not show any minimum and, therefore, the minimum in the linewidth data is related to the external field required for resonance. The ESR parameters show no anomalies around $T=T_{\mathrm{C}}$. This agrees with the previous results [8, 13] and is consistent with the smooth variation of the magnetization across $T_{\mathrm{C}}$. In addition, there is no extraordinary behavior of the ESR parameters for the Lu-substituted samples near the QCP at $x_{\mathrm{c}}=0.035$. All these samples show a continuous decrease of the linewidth towards low temperatures, as similarly found in the quantum critical material YbRh$_{2}$Si$_{2}$ doped with Ge or La [16, 17].

We interpret the linewidth data in terms of the relaxation of a conduction spin resonance (CESR) in itinerant magnets such as ZrZn$_{2}$, TiBe$_{2}$, or NbFe$_{2}$ [3–5, 13]. The spin–lattice relaxation which leads to the CESR linewidth is due to the spin–orbit coupling to transport collisions and can be related to the collision time *via* the g value-shift (Elliott–Yafet theory [14, 15]). In exchange coupled spin-systems, the spin-lifetimes are further enhanced by the exchange interaction [7]. The relaxation is then also determined by the internal exchange field. In the limit of a much larger exchange field than the applied field, the linewidth should be inversely proportional to the magnetization.

Looking at the general linewidth behavior of the CESR in (Sc$_{1-x}$Lu$_{x}$)$_{3.1}$In, the increase with temperature is determined by the inverse magnetization (see Fig. 2 in Ref. [1]). The Lu-substituted samples show larger linewidths because of their much smaller magnetisation and the decreasing Curie–Weiss-*like* temperature $T^{*}_{\mathrm{C}}$ [1], which is a measure of ferromagnetic correlations. Hence, the exchange enhancement of the spin-lifetimes is less effective, leading to a considerable linewidth contribution of the enhanced spin fluctuations due to disorder, brought on by Lu substitution. For the Lu-substituted samples, one finds a relation between the resistivity (expressed as the residual resistivity ratio RRR) and the residual linewidth (i.e., the linewidth obtained by linearly extrapolating the data towards zero temperature). The smaller the 1/RRR (i.e., the smaller the disorder) the smaller the residual, zero-temperature linewidth. This indicates that disorder, introduced by Lu substitution, is also an important ingredient for the relaxation mechanism of the observed resonance. This was similarly discussed for the ESR in the La-substituted Kondo lattice system YbRh$_{2}$Si$_{2}$ [16], where the resonance is understood to originate from the formation of collective spin modes of Kondo ions and conduction electrons [18].

The resonance field varies only weakly with temperature and there is no clear feature seen around $T_{\mathrm{C}}$. For $T=5$ K, an ESR g value of $2.01\pm 0.01$ (as calculated from the resonance condition $g\mu _{\mathrm{B}}B_{\mathrm{res}}=h\nu$) characterizes the spectra of all investigated (Sc$_{1-x}$Lu$_{x}$)$_{3.1}$In compounds.

## Conclusion

The ESR in (Sc$_{1-x}$Lu$_{x}$)$_{3.1}$In shows a typical Dysonian lineshape for all investigated Lu concentrations which establishes itinerant resonant spins. Their relaxation behavior is consistent within a picture for exchange coupled spin-systems, i.e., the linewidth is determined by the inverse of the magnetization.

## References

[1] Svanidze E, Liu L, Frandsen B, White BD, Besara T, Goko T, Medina T, Munsie TJS, Luke GM, Zheng D, Jin CQ, Siegrist T, Maple MB, Uemura YJ, Morosan E (2015). Phys. Rev. X.

[2] Moriya T (1985). Spin Fluctuations in Itinerant Electron Magnetism.

[3] Shaltiel D (1988). Helv. Phys. Acta.

[4] Förster T, Sichelschmidt J, Grüner D, Brando M, Kimura N, Steglich F (2010). J. Phys. Conf. Ser..

[5] Rauch D, Kraken M, Litterst FJ, Süllow S, Luetkens H, Brando M, Förster T, Sichelschmidt J, Neubauer A, Pfleiderer C, Duncan WJ, Grosche FM (2015). Phys. Rev. B.

[6] Brinkman WF, Engelsberg S (1968). Phys. Rev. Lett..

[7] Fulde P, Luther A (1968). Phys. Rev..

[8] Dunifer GL, Knapp GS, Corenzwit E (1970). J. Appl. Phys..

[9] Joshi J, Bhat S (2004). J. Magn. Reson..

[10] Feher G, Kip AF (1955). Phys. Rev..

[11] Dyson F (1955). Phys. Rev..

[12] Lesseux GG, Garitezi TM, Rosa PFS, Jesus CBR, Oseroff SB, Sarrao JL, Fisk Z, Urbano RR, Pagliuso PG, Rettori C (2016). J. Phys. Condens. Matter.

[13] Walsh WM, Knapp GS, Rupp JLW, Schmidt PH (1970). J. Appl. Phys..

[14] Elliott RJ (1954). Phys. Rev..

[15] Yafet Y (1952). Phys. Rev..

[16] Wykhoff J, Sichelschmidt J, Ferstl J, Krellner C, Geibel C, Steglich F, Fazlishanov I, Krug von Nidda HA (2007). Phys. C.

[17] Sichelschmidt J, Ferstl J, Geibel C, Steglich F (2005). Phys. B.

[18] Kochelaev BI (2017). Low Temp. Phys..

